# Identifying prognostic factors for clinical outcomes and costs in four high-volume surgical treatments using routinely collected hospital data

**DOI:** 10.1038/s41598-022-09972-6

**Published:** 2022-04-07

**Authors:** N. Salet, V. A. Stangenberger, F. Eijkenaar, F. T. Schut, M. C. Schut, R. H. Bremmer, A. Abu-Hanna

**Affiliations:** 1 Erasmus School of Health Policy and Management, Erasmus University, Rotterdam, The Netherlands; 2 Department of Medical Informatics, Amsterdam UMC, University of Amsterdam, Amsterdam, The Netherlands; 3 LOGEX b.v., Amsterdam, The Netherlands

**Keywords:** Cardiology, Gastroenterology, Medical research, Risk factors, Urology, Health care, Prognosis, Oncology, Surgical oncology

## Abstract

Identifying prognostic factors (PFs) is often costly and labor-intensive. Routinely collected hospital data provide opportunities to identify clinically relevant PFs and construct accurate prognostic models without additional data-collection costs. This multicenter (66 hospitals) study reports on associations various patient-level variables have with outcomes and costs. Outcomes were in-hospital mortality, intensive care unit (ICU) admission, length of stay, 30-day readmission, 30-day reintervention and in-hospital costs. Candidate PFs were age, sex, Elixhauser Comorbidity Score, prior hospitalizations, prior days spent in hospital, and socio-economic status. Included patients dealt with either colorectal carcinoma (CRC, n = 10,254), urinary bladder carcinoma (UBC, n = 17,385), acute percutaneous coronary intervention (aPCI, n = 25,818), or total knee arthroplasty (TKA, n = 39,214). Prior hospitalization significantly increased readmission risk in all treatments (OR between 2.15 and 25.50), whereas prior days spent in hospital decreased this risk (OR between 0.55 and 0.95). In CRC patients, women had lower risk of in-hospital mortality (OR 0.64), ICU admittance (OR 0.68) and 30-day reintervention (OR 0.70). Prior hospitalization was the strongest PF for higher costs across all treatments (31–64% costs increase/hospitalization). Prognostic model performance (c-statistic) ranged 0.67–0.92, with Brier scores below 0.08. R-squared ranged from 0.06–0.19 for LoS and 0.19–0.38 for costs. Identified PFs should be considered as building blocks for treatment-specific prognostic models and information for monitoring patients after surgery. Researchers and clinicians might benefit from gaining a better insight into the drivers behind (costs) prognosis.

## Introduction

Predicting the course of disease and outcome of treatment is crucial for both physicians and patients. Prognostic factor (PF) research is a fundamental first step^1^ in developing accurate prognostic models for that purpose. PFs are defined as measures that are available at the time of diagnosis, and that are associated with a subsequent clinical outcome^2^. PF research plays a crucial role in many areas that are relevant to clinical practice, including establishing treatment options, identifying targets for intervention, supporting shared decision-making, and providing more affordable methods for prognosis.

A recent review of PF studies has identified several limitations in PF research, including insufficient sample size, inappropriate analyses, and unclear reporting^2^. Furthermore, PF research often lacks standardized adjustment for comorbidity, even though this is likely to generate more accurate and generalizable results. Another limitation relates to (the high costs associated with) data availability and translating those data into relevant information. In PF research, data are typically collected and processed in a labor-intensive manner, requiring a substantial number of resources. This is particularly true for biomarkers^3^, which are often unavailable and/or disproportionately costly to collect and in addition, organic materials often have limited longevity^4^. Using routinely collected hospital data for PF research might present cost-effective opportunities to contribute to knowledge about which patient factors influence outcomes and costs. In turn, identified PFs might be added to (existing) prognostic models to further improve individualized risk prediction.

The premise of this paper is that routinely collected data in hospital information systems may be of significant value in PF identification and the subsequent construction of prognostic models, which could in turn yield clinically relevant information. Hospital information systems mainly contain electronic health records (EHR) and billing/reimbursement data and are one of the fastest-growing data sources in health care. In addition, prior research has underlined the potential of these data for improving the value (i.e. the outcomes achieved at given level of costs) of healthcare delivery^5–7^. More specifically, by providing insight into patients’ health status (e.g. survival), recovery process (e.g. complications) and sustainability of health (e.g. readmission), these data form a potentially useful source for reliable costs and outcome measurements, which could in turn be used for various methods for steering on value^8^. Furthermore, these data typically allow for the retrieval of secondary diagnoses, which enables standardized comorbidity adjustment.

The primary objective of this study is to investigate to what extent is it possible to identify (common) PF associations with outcomes and costs across four high-volume surgical treatments, using routinely collected data from 66 Dutch hospitals. More specifically, we investigate possible associations of various candidate-PFs with five clinical outcomes and in-hospital costs. The secondary objective is to evaluate the discriminative ability and predictive accuracy of prognostic models in which we combine the identified PFs.

## Results

### Descriptive statistics

**Figure 1:**
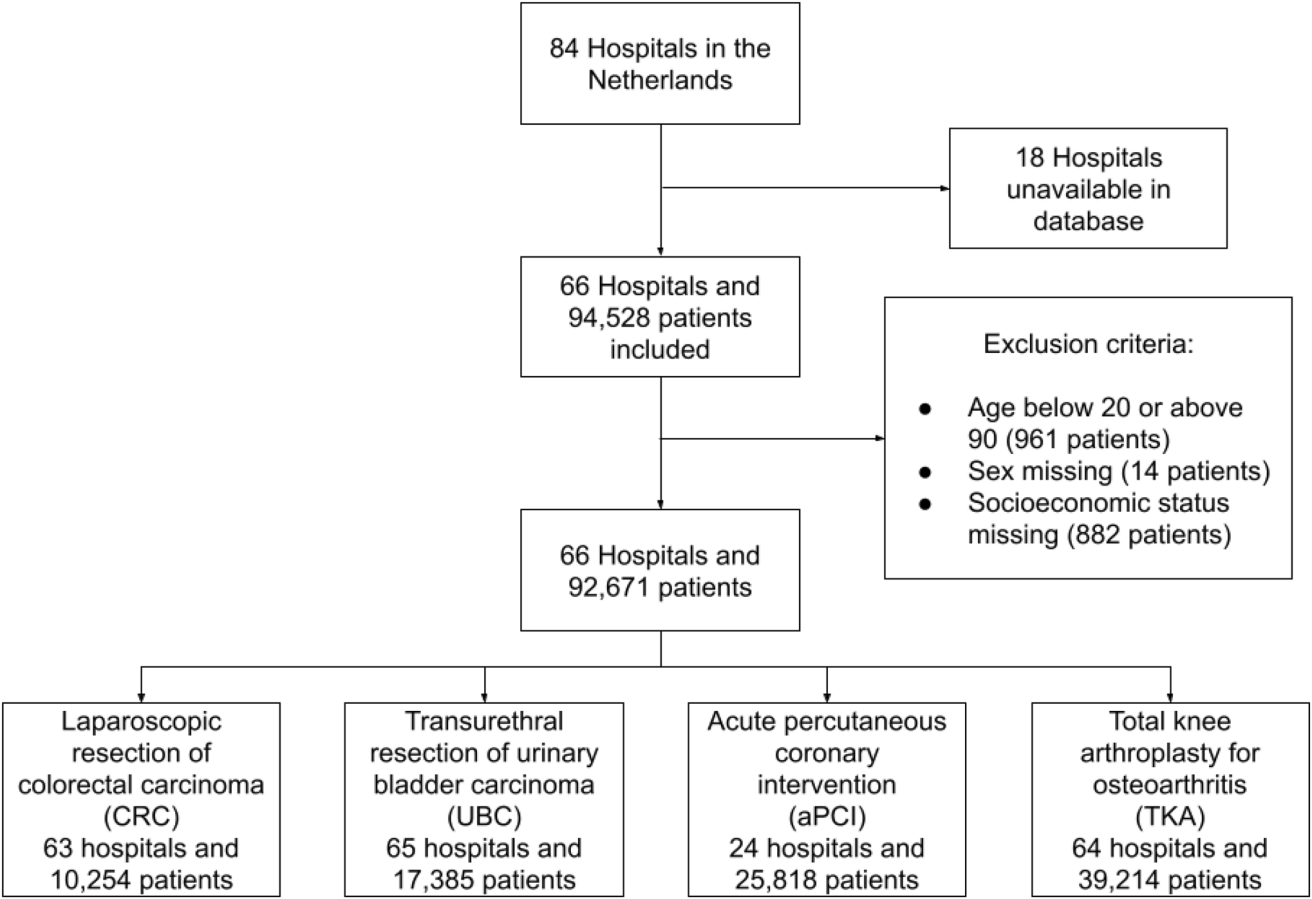
Flowchart describing study population and treatments.

In total, 92,671 patients treated in 66 Dutch hospitals over a two-year period (2016–2017) were included (Fig. 1). Patients in this cohort received one of the four abovementioned surgical interventions: CRC (n = 10,254), UBC (n = 17,385), aPCI (n = 25,818), and TKA (n = 39,214). The mean age of the cohort was 68.1 years, and 44.7% of the patients were female (Table 1). Patients with CRC and UBC suffered from more severe comorbidity, translating into higher Elixhauser Comorbidity Scores (ECSs): 4.9 and 5.5 for UBC and CRC patients versus 1.2 and 1.1 for aPCI and TKA patients. In-hospital mortality was higher in aPCI patients (2.2%) than in patients with other conditions (0.1–1.4%). ICU admission rates were the lowest in TKA patients (0.8%) and the highest in CRC patients (10.4%). The median LoS after surgery was the highest in CRC patients (5 days) and the lowest in UBC patients (1 day). By contrast, readmission (7.6%) and reintervention (3.7%) rates were highest in UBC patients. CRC was the most expensive treatment with a median total cost of €11,707, followed by TKA (€9251), aPCI (€4984) and UBC (€4721) (Table 2).

**Table 1:** Overview of study population and summary statistics of candidate PF variables, by surgical treatment.

Variable	Laparoscopic resection of colorectal carcinoma (CRC)	Transurethral resection of urinary bladder carcinoma (UBC)	Acute percutaneous coronary intervention (aPCI)	Total knee arthroplasty for osteoarthritis (TKA)	Total study population
Total number of patients	10,254	17,385	25,818	39,214	92,671
Number of hospitals	63	65	24	64	66
Number of patients in 2016 (% of total)	5566 (54.3%)	9911 (57.0%)	13,204 (51.1%)	20,765 (53.0%)	49,446 (53.4%)
Number of patients in 2017 (% of total)	4688 (45.7%)	7474 (43.0%)	12,614 (48.9%)	18,449 (47.0%)	43,225 (46.6%)
Mean age (SD)	67.4 (12.7)	70.8 (11.0)	65.1 (12.0)	69.0 (9.2)	68.1 (11.0)
Female sex (%)	5028 (49.0%)	4114 (23.7%)	7229 (28.0%)	25,043 (63.9%)	41,414 (44.7%)
Elixhauser index 0 (%)	1094 (10.7%)	731 (4.2%)	19,990 (77.4%)	29,092 (74.2%)	50,907 (54.9%)
Elixhauser index 1 (%)	6219 (60.6%)	1,0879 (62.6%)	3892 (15.1%)	7594 (19.4%)	28,584 (30.8%)
Elixhauser index 2 (%)	1780 (17.4%)	4015 (23.1%)	1224 (4.7%)	1878 (4.8%)	8897 (9.6%)
Elixhauser index 3 (%)	673 (6.6%)	1254 (7.2%)	459 (1.8%)	469 (1.2%)	2855 (3.1%)
Elixhauser index 4 (%)	317 (3.1%)	374 (2.2%)	118 (0.5%)	132 (0.3%)	941 (1.0%)
Elixhauser index 5 (%)	109 (1.1%)	80 (0.5%)	86 (0.3%)	39 (0.1%)	314 (0.3%)
Elixhauser index > 5 (%)	62 (0.6%)	52 (0.3%)	49 (0.2%)	10 (0.0%)	173 (0.2%)
Mean Elixhauser comorbidity score (SD)	4.9 (3.8)	5.5 (3.8)	1.2 (3.1)	1.1 (2.8)	2.3 (3.7)
Socio-Economic Status class 1 (%) *reference*	3306 (32.2%)	5298 (30.5%)	6739 (26.1%)	11,559 (29.5%)	26,902 (29.0%)
Socio-Economic Status class 2 (%)	3767 (36.7%)	6214 (35.7%)	9125 (35.3%)	14,820 (37.8%)	33,926 (36.6%)
Socio-Economic Status class 3 (%)	3181 (31.0%)	5873 (33.8%)	9954 (38.6%)	12,835 (32.7%)	31,843 (34.4%)
0 hospitalizations in years prior to treatment (%)	9597 (93.6%)	16,358 (94.1%)	25,747 (99.7%)	38,881 (99.2%)	90,583 (97.7%)
1 hospitalization in years prior to treatment (%)	554 (5.4%)	833 (4.8%)	67 (0.3%)	314 (0.8%)	1768 (1.9%)
2 hospitalizations in years prior to treatment (%)	81 (0.8%)	137 (0.8%)	3 (0.0%)	16 (0.0%)	237 (0.3%)
> 2 hospitalizations in years prior to treatment (%)	22 (0.2%)	41 (0.3%)	1 (0.0%)	3 (0.0%)	83 (0.1%)

**Table 2:** Summary statistics of outcomes and costs, by surgical treatment.

Variable	Shown as	Laparoscopic resection of colorectal carcinoma (CRC)	Transurethral resection of urinary bladder carcinoma (UBC)	Acute percutaneous coronary intervention (aPCI)	Total knee arthroplasty for osteoarthritis (TKA)	Total study population
In-hospital mortality	n (%)	148 (1.4%)	179 (1.0%)	570 (2.2%)	39 (0.1%)	936 (1.0%)
ICU admittance	n (%)	1065 (10.4%)	298 (1.7%)	1220 (4.7%)	298 (0.8%)	2881 (3.1%)
Length of stay after intervention	median [IQR]	5.0 [4.0; 7.0]	1.0 [1.0; 1.0]	2.0 [0.0; 3.0]	2.0 [2.0; 3.0]	2.0 [1.0; 3.0]
Readmission within 30 days	n (%)	440 (4.3%)	1314 (7.6%)	99 (0.4%)	676 (1.7%)	2549 (2.8%)
Reintervention within 30 days	n (%)	13 (0.1%)	647 (3.7%)	266 (1.0%)	61 (0.2%)	1013 (1.1%)
Costs additional	€ median [IQR]	0.0 [0.0; 0.0]	0.0 [0.0; 0.0]	593.0 [593.0; 1187.0]	0.0 [0.0; 0.0]	0.0 [0.0; 248.0]
Costs clinic	€ median [IQR]	3017.0 [2193.0; 4779.0]	1364.0 [1008.0; 2436.0]	1549.0 [773.0; 2551.0]	1399.0 [1399.0; 2303.0]	1752.0 [1048.0; 2551.0]
Costs consultation	€ median [IQR]	388.0 [298.0; 551.0]	314.0 [202.0; 497.0]	139.0 [0.0; 351.0]	190.0 [79.0; 269.0]	228.0 [82.0; 351.0]
Costs diagnostic	€ median [IQR]	765.0 [478.0; 1178.0]	673.0 [420.0; 1064.0]	727.0 [322.0; 1183.0]	166.0 [101.0; 260.0]	353.0 [153.0; 824.0]
Costs surgical	€ median [IQR]	7367.0 [7367.0; 7367.0]	1515.0 [1515.0; 3030.0]	1558.0 [1558.0; 1558.0]	7047.0 [7047.0; 7047.0]	4951.0 [1558.0; 7047.0]
Costs total	€ median [IQR]	11,707.0 [10,579.0; 14,869.2]	4721.0 [3498.0; 7680.0]	4984.0 [3874.0; 6442.0]	9251.0 [8640.0; 10,326.0]	8555.0 [5127.5; 10,241.5]

### Main results

Across the four treatments and six outcomes (including costs), we identified numerous statistically significant PF associations (Tables 3, 4, 5, 6). Notable differences were also identified between individual treatments and cohort results (Appendix 1). Below, however, we will limit the presentation to the results expected to have the highest clinical relevance. Results are presented by outcome with reference to corresponding treatments. This section ends with our findings on prognostic model performance.

**Table 3:** Prognostic factors for outcomes and costs for colorectal carcinoma, where *p ≤ 0.05.

	In-hospital mortality, odds ratio (95% CI)	Readmission, odds ratio (95% CI)	Reintervention, odds ratio (95% CI)	ICU admission, odds ratio (95% CI)	Length of stay, coefficient (95% CI)	Total in-hospital costs, coefficient (95% CI)
**Laparoscopic resection of colorectal carcinoma (CRC)**						
Age	1.10* (1.08; 1.13)	1.00 (0.99; 1.00)	1.00 (0.98; 1.01)	1.03* (1.03; 1.04)	0.00* (0.00; 0.00)	1.002* (1.002; 1.003)
Female sex	0.64* (0.45; 0.90)	1.00 (0.81; 1.25)	0.70* (0.49; 0.99)	0.68* (0.59; 0.78)	− 0.03* (− 0.04; − 0.01)	0.973* (0.960; 0.987)
Elixhauser Comorbidity score	1.05* (1.02; 1.09)	1.04* (1.01; 1.06)	1.07* (1.03; 1.11)	1.10* (1.08; 1.11)	0.01* (0.01; 0.01)	1.013* (1.011; 1.015)
# Hospitalizations in 365 days prior to treatment	1.73* (1.16; 2.47)	25.50* (19.63; 33.30)	1.63* (1.09; 2.31)	1.89* (1.60; 2.24)	0.27* (0.25; 0.29)	1.310* (1.279; 1.342)
# Days in hospital in 365 days prior to treatment	1.02 (0.96; 1.09)	0.67* (0.62; 0.72)	1.02 (0.96; 1.09)	1.01 (0.98; 1.04)	0.03* (0.02; 0.03)	1.029* (1.025; 1.032)
SES 2	1.29 (0.85; 1.96)	1.22 (0.93; 1.61)	1.17 (0.75; 1.81)	1.15 (0.96; 1.37)	0.01 (− 0.01; 0.02)	1.007 (0.990; 1.024)
SES 3	1.36 (0.88; 2.10)	1.40* (1.05; 1.86)	1.48 (0.95; 2.29)	1.31* (1.08; 1.58)	0.03* (0.01; 0.04)	1.027* (1.007; 1.046)

**Table 4:** Prognostic factors for outcomes and costs for urinary bladder carcinoma, where *p ≤ 0.05.

	In-hospital mortality, odds ratio (95% CI)	Readmission, odds ratio (95% CI)	Reintervention, odds ratio (95% CI)	ICU admission, odds ratio (95% CI)	Length of Stay, coefficient (95% CI)	Total in-hospital costs, coefficient (95% CI)
**Transurethral resection of urinary bladder carcinoma (UBC)**						
Age	1.06* (1.04; 1.08)	1.01* (1.00; 1.01)	0.99 (0.99; 1.00)	1.00 (0.99; 1.01)	0.00* (0.00; 0.00)	1.002* (1.002; 1.003)
Female sex	1.52* (1.09; 2.13)	0.73* (0.63; 0.85)	0.74* (0.60; 0.91)	0.88 (0.65; 1.18)	− 0.08* (− 0.10; − 0.06)	0.920* (0.903; 0.938)
Elixhauser Comorbidity score	1.06* (1.03; 1.09)	1.02* (1.01; 1.04)	1.02 (1.00; 1.04)	1.08* (1.06; 1.11)	0.01* (0.01; 0.01)	1.001* (1.001; 1.012)
# Hospitalizations in 365 days prior to treatment	0.87 (0.62; 1.20)	2.15* (1.87; 2.49)	1.53* (1.27; 1.84)	1.42* (1.18; 1.72)	0.28* (0.26; 0.31)	1.328* (1.294; 1.363)
# Days in hospital in 365 days prior to treatment	1.16* (1.11; 1.22)	0.95* (0.91; 0.99)	0.93* (0.87; 1.00)	1.07* (1.03; 1.12)	0.07* (0.07; 0.08)	1.075* (1.069; 1.081)
SES 2	1.25 (0.84; 1.86)	1.18* (1.02; 1.37)	1.04 (0.85; 1.28)	0.99 (0.74; 1.34)	0.01 (− 0.01; 0.03)	1.001 (0.987; 1.028)
SES 3	1.40 (0.94; 2.08)	1.14 (0.97; 1.33)	1.12 (0.90; 1.39)	0.93 (0.68; 1.27)	0.01 (− 0.01; 0.03)	1.007 (0.986; 1.029)

**Table 5:** Prognostic factors for outcomes and costs for acute coronary intervention, where *p ≤ 0.05.

	In-hospital mortality, odds ratio (95% CI)	Readmission, odds ratio (95% CI)	Reintervention, odds ratio (95% CI)	ICU admission, odds ratio (95% CI)	Length of stay, coefficient (95% CI)	Total in-hospital costs, coefficient (95% CI)
**Acute percutaneous coronary intervention (aPCI)**						
Age	1.04* (1.04; 1.05)	1.01 (0.99; 1.03)	1.00 (0.99; 1.01)	1.00 (0.99; 1.00)	0.00* (0.00; 0.00)	1.002* (1.002; 1.002)
Female sex	1.16 (0.97; 1.39)	0.46* (0.26; 0.80)	0.79 (0.59; 1.05)	0.89 (0.78; 1.02)	− 0.02* (− 0.03; − 0.01)	1.006* (1.002; 1.010)
Elixhauser Comorbidity score	1.02* (1.00; 1.05)	1.05 (0.99; 1.11)	1.03 (0.99; 1.06)	1.08* (1.07; 1.10)	0.02* (0.02; 0.03)	1.005* (1.004; 1.006)
# Hospitalizations in 365 days prior to treatment	1.57 (0.37; 3.71)	25.44* (8.80; 68.84)	7.83* (3.91; 15.24)	1.93* (1.01; 3.69)	0.25* (0.16; 0.35)	1.635* (1.601; 1.670)
# Days in hospital in 365 days prior to treatment	0.78* (0.69; 0.87)	0.61* (0.40; 0.92)	0.98 (0.91; 1.06)	1.04* (1.01; 1.07)	0.08* (0.08; 0.08)	1.033* (1.030; 1.037)
SES 2	1.13 (0.91; 1.42)	0.72 (0.44; 1.19)	1.03 (0.75; 1.43)	1.04 (0.89; 1.21)	− 0.00 (− 0.02; 0.01)	1.007* (1.002; 1.012)
SES 3	1.03 (0.82; 1.31)	0.74 (0.44; 1.25)	1.06 (0.77; 1.47)	0.99 (0.84; 1.16)	− 0.01 (− 0.02; 0.01)	1.014* (1.009; 1.020)

**Table 6:** Prognostic factors for outcomes and costs for knee osteoarthritis, where *p ≤ 0.05.

	In-hospital mortality, odds ratio (95% CI)	Readmission, odds ratio (95% CI)	Reintervention, odds ratio (95% CI)	ICU admission, odds ratio (95% CI)	Length of stay, coefficient (95% CI)	Total in-hospital costs, coefficient (95% CI)
**Total knee arthroplasty for osteoarthritis (TKA)**						
Age	1.09* (1.04; 1.13)	1.02* (1.01; 1.03)	0.97* (0.95; 1.00)	1.00 (0.98; 1.01)	0.00* (0.00; 0.00)	1.002* (1.002; 1.002)
Female sex	0.82 (0.43; 1.57)	0.67* (0.57; 0.78)	0.72 (0.43; 1.19)	0.56* (0.44; 0.71)	0.01* (0.00; 0.01)	1.006* (1.002; 1.010)
Elixhauser Comorbidity score	1.16* (1.11; 1.22)	1.08* (1.06; 1.10)	0.99 (0.90; 1.10)	1.18* (1.16; 1.21)	0.00* (0.00; 0.01)	1.005* (1.004; 1.006)
# Hospitalizations in 365 days prior to treatment	1.33 (0.02; 5.42)	23.40* (15.67; 35.18)	3.60 (0.86; 8.09)	1.54 (0.68; 3.08)	0.49* (0.47; 0.51)	1.635* (1.601; 1.670)
# Days in hospital in 365 days prior to treatment	1.08 (0.93; 1.24)	0.55* (0.43; 0.70)	0.68 (0.25; 1.81)	1.03 (0.93; 1.16)	0.03* (0.03; 0.04)	1.033* (1.030; 1.037)
SES 2	0.83 (0.40; 1.73)	1.15 (0.95; 1.40)	0.84 (0.48; 1.47)	1.41* (1.03; 1.92)	0.01* (0.00; 0.01)	1.007* (1.002; 1.012)
SES 3	0.62 (0.27; 1.40)	1.16 (0.94; 1.43)	0.52 (0.25; 1.09)	1.47* (1.05; 2.06)	0.01* (0.01; 0.02)	1.014* (1.009; 1.020)

### In-hospital mortality

In CRC patients, age (OR 1.10), ECS (OR 1.05) and prior hospitalizations (OR 1.73) were significantly associated with a higher risk of in-hospital mortality. In addition, women had a significantly lower mortality risk than men (OR 0.64).

In UBC patients, age (OR 1.10), ECS (OR 1.06), female sex (OR 1.52) and prior days spent in hospital (OR 1.16) were significantly associated with in-hospital mortality risk.

In aPCI patients, age (OR 1.04) and ECS (OR 1.02) were found to be statistically significant PFs for higher in-hospital mortality. By contrast, prior days spent in hospital days (OR 0.78) were significantly associated with reduced risk of in-hospital mortality.

Finally, age (OR 1.09) and ECS (OR 1.16) were also found to be positively associated with this outcome for TKA patients.

### ICU admission

For CRC patients, statistically significant associations with a higher risk of ICU admission were found for age (OR 1.03), ECS (OR 1.10), prior hospitalizations (OR 1.89) and low SES (OR 1.31, compared to high SES). By contrast, female sex significantly reduces this risk (OR 0.68).

In UBC patients, ECS (OR 1.08), prior hospitalizations (OR 1.42) and prior days spent in hospital (OR 1.07) were positively related to the risk of ICU admission.

Both ECS (OR 1.08) and prior hospitalizations (OR 1.93) significantly increased ICU admission risk in patients undergoing aPCI.

ECS (OR 1.18), medium SES (1.41) and low SES (OR 1.47) were found to be significantly associated with an increased risk of this outcome in TKA patients, whereas a negative association was found for female sex (OR 0.56).

### 30-day readmission

In CRC patients, prior hospitalizations (OR 25.50) were associated with an increased readmission risk, as were ECS (OR 1.04) and low SES (OR 1.40).

In UBC patients, age (OR 1.01), ECS (OR 1.02), and prior hospitalizations (OR 2.15) were identified as statistically significant PFs for increased readmission risk. Female sex (OR 0.73) and prior days spent in hospital (OR 0.95) were negatively associated with this outcome for this patient group.

Prior hospitalizations were strongly associated (OR 25.44) with increased readmission risk in aPCI patients, while we found the opposite for the variables female sex (OR 0.46) and prior days spent in hospital (OR 0.61).

In TKA patients, age (OR 1.02), ECS (OR 1.08), and prior hospitalizations (OR 23.40) were positively associated with the risk of this outcome. Again, we found an association with the opposite direction for female sex (OR 0.67) and prior days spent in hospital (OR 0.55).

### 30-day reintervention

In CRC patients, we found ECS (OR 1.07) and prior hospitalizations (OR 1.63) to be significantly associated with an increased reintervention risk, while a negative association was found again for female sex (OR 0.70).

Similar results were found for UBC patients, with a positive association for prior hospitalizations (OR 1.53) and a negative association for female sex (OR 0.74). In addition, for this patient group we also found a (weak) negative association between prior days spent in hospital and this outcome (OR 0.93).

Also, among aPCI patients, prior hospitalizations (OR 7.83) were associated with a higher reintervention risk.

Finally, only age (OR 1.02) was identified as a PF in TKA patients for this outcome.

### Length of stay

For length of stay, a significant positive effect was found for prior hospitalizations among aPCI patients (*b* 0.25), CRC patients (*b* 0.27), UBC patients (*b* 0.28) and TKA patients (*b* 0.49).

### In-hospital costs

Prior hospitalizations were most strongly associated with costs for aPCI patients, with an estimated average cost increase of 63% per additional prior hospitalization, all else equal. Positive associations were also found for patients who underwent CRC (31%), UBC (32%), or TKA (33%). Female sex was negatively associated with costs for CRC (− 2.7%) and UBC patients (− 8.0%). Finally, prior days spent in hospital were identified as a PF for higher costs, with the estimated effect ranging from 2.9% (CRC patients) to 7.5% (UBC patients) average costs increase per additional day in hospital prior to treatment.

### Prognostic model performance

Subsequently, the discriminative ability, predictive accuracy and model fit statistics of prognostic models was evaluated (Tables 7, 8).

**Table 7:** Model fit statistics and brier score for dichotomous outcomes.

	Mortality	Readmission	Reintervention	ICU admittance
**Laparoscopic resection of colorectal carcinoma (CRC)**				
C-statistic (CI)	0.84 (0.84; 0.89)	0.85 (0.84; 0.88)	0.74 (0.74–0.84)	0.78 (0.78; 0.81)
Brier score	0.01	0.03	0.01	0.08
**Transurethral resection of urinary bladder carcinoma (UBC)**				
C-statistic (CI)	0.81 (0.81; 0.86)	0.67 (0.67; 0.70)	0.71 (0.71; 0.75)	0.79 (0.79; 0.83)
Brier score	0.01	0.07	0.04	0.02
**Acute percutaneous coronary intervention (aPCI)**				
C-statistic (CI)	0.77 (0.75; 0.80)	0.82 (0.80; 0.88)	0.67 (0.66; 0.72)	0.68 (0.67; 0.70)
Brier score	0.02	0.00	0.01	0.04
**Total knee arthroplasty for osteoarthritis (TKA)**				
C-statistic (CI)	0.92 (0.92; 0.97)	0.71 (0.71; 0.74)	0.90 (0.90; 0.95)	0.88 (0.88; 0.91)
Brier score	0.00	0.02	0.00	0.00

**Table 8:** Model fit statistics for continuous outcomes.

	Length of stay	Hospital costs
**Laparoscopic resection of colorectal carcinoma (CRC)**		
R-squared	0.06	0.26
**Transurethral resection of urinary bladder carcinoma (UBC)**		
R-squared	0.10	0.21
**Acute percutaneous coronary intervention (aPCI)**		
R-squared	0.07	0.19
**Total knee arthroplasty for osteoarthritis (TKA)**		
R-squared	0.19	0.38

In CRC patients, c-statistic values were 0.84 (CI 0.84–0.89) for in-hospital mortality, 0.78 (CI 0.78–0.81) for ICU admittance, 0.85 (CI 0.84–0.88) for readmission, and 0.74 (CI 0.74–0.84) for reintervention, suggesting fair to good discriminative ability. The R-squared for LoS was 0.06 and 0.26 for costs.

In the UBC patients, the c-statistic also varied across models: 0.81 (CI 0.81–0.86) for in-hospital mortality, 0.79 (CI 0.79–0.83) for ICU admittance, 0.67 (CI 0.67–0.70) for readmission, and 0.71 (CI 0.71–0.75) for reintervention. The R-squared for LoS was 0.10 and 0.21 for costs.

In patients who underwent aPCI, c-statistics were 0.77 (CI 0.75–0.80) for in-hospital mortality, 0.68 (CI 0.67–0.70) for ICU admittance, 0.82 (CI 0.80–0.88) for readmission, and 0.67 (CI 0.66–0.72) for reintervention. The R-squared for LoS was 0.07 and 0.19 for costs.

In TKA patients, c-statistics were 0.92 (CI 0.92–0.97) for in-hospital mortality, 0.88 (CI 0.88–0.91) for ICU admittance, 0.71 (CI 0.71–0.74) for readmission, and 0.90 (CI 0.90–0.95) for reintervention. The R-squared for LoS was 0.19 and 0.38 for costs.

Finally, across the models for dichotomous outcomes, the Brier score was consistently below 0.08, suggesting good to excellent predictive accuracy.

## Discussion

Using data that are routinely available in hospital information systems, this study has generated clinically relevant knowledge on PFs for five outcomes as well as in-hospital costs in four high-volume surgical treatments. The PFs that influenced clinical outcomes most across all treatments were sex, comorbidity and prior hospitalizations. The latter PF was also most strongly predictive of costs. Constructed prognostic models achieved fair to excellent discriminative abilities and had low Brier scores, underlining the potential of using routinely collected data for PF research. Although the proportion of variance in LoS that was explained by our model is limited, clinicians and policy makers might find the explained proportion of costs variance insightful because these highlight targets for costs reduction strategies through interventions that reduce costs variation^9,10^.

Across the surgical interventions analyzed, we identified several common PFs for outcomes and costs. Given that these PFs were identified across four distinct treatments, similar associations may well exist for other (surgical) treatments too. Although originally validated as a prognostic tool for in-hospital mortality, the ECS might have wider applicability^11^. Apart from readmission risk in aPCI patients, the ECS was found to be a PF for increased risk of ICU admission and of 30-day readmission, as well as higher LoS. In addition, prior hospitalizations were identified as a strong PF for increased readmission risk across all treatments. This association was previously identified in a non-surgical setting^12^. In contrast, prior days spent in hospital was associated with lower readmission risk. Although longer LoS after surgery was associated with decreased readmission risk in other surgical treatments^13,14^, we did not encounter work that previously identified or described the association between (all-cause) prior days spent in hospital and decreased readmission risk.

Finally, prior hospitalizations were strongly and positively associated with costs across all treatments. Given this, and the strong (intermediary) association that prior hospitalizations and readmission risk have, increased spending on readmission prevention could result in a net costs saving for these treatments^15^.

Comparison of the results for the cohort to those for the underlying treatment subgroups suggests that PF research could benefit from differentiating between specific (surgical) treatments**.** To illustrate, there has been debate on whether age should always be considered when determining the risk of ICU admission^16^. We found age to be a PF for ICU admission risk in some treatments, but not all. A similar argument can be made for age in relation to readmission and reintervention risk. Moreover, we sometimes encountered markedly divergent results across outcomes in terms of statistical significance of PF associations when models were estimated on the cohort instead of separately for the four distinct treatments. In short, PFs should be identified for specific combinations of target condition and (surgical) intervention. Ideally, these models should include standardized comorbidity adjustment, which can be done using routinely collected hospital data, as we have shown.

To our knowledge, this is the first study that identified multiple PFs for five outcomes and costs across four different surgical treatments using routinely collected hospital data. Among the strengths of this study are its large sample size and its multicenter design. Due to its national character and the underlying automation of the routine data collection process, risks (selection and attrition bias) often associated with observational studies are unlikely to have meaningfully distorted our results. Identified PFs both represent new knowledge and confirm or contradict PFs identified in previous work (e.g. female sex was found to be associated with far lower readmission risk for aPCI treatments, in contrast to earlier research focusing on non-acute infarctions^17^). In addition, we believe that it should be possible to reproduce our approach of repurposing routinely collected data for PF research for many other (surgical) treatments. Future work in PF research per our approach might further expand clinical knowledge by focusing research questions on different treatments, comparing treatment options, intercountry differences and or using existing registries more efficiently.

Some limitations intrinsic to the study design should also be mentioned. First, although our data allowed for the measurement and analysis of several clinically relevant candidate PFs, our results may have been influenced by the effect of unobserved confounding (e.g., clinical factors such as disease progression and complexity, and lifestyle-related factors like smoking). For example, while SES is known to be associated with smoking^18^ and might also play a role in obesity^19^, we were unable to adjust for this due to lack of data. Data on these factors often is of poor quality due to factors such as incomplete registration^20,21^. Second, the generalizability of our results might be influenced by contextual factors (e.g., treatment country, surgeon performance, hospital characteristics, surgical approach, and hospital/surgeon volume) underlining the importance of future studies in other countries and settings. Third, although highly unlikely, due to privacy regulations we cannot preclude the possibility of patients having received additional treatment from a different hospital during their initial treatment, which may have resulted in an underestimation of adverse events. Another limitation is that although one-year follow-up often includes the entirety of hospital treatment, we have no record of longer-term outcomes or costs. Finally, although inhospital mortality, ICU admission and 30-day readmission can be considered proxy-outcomes for complications, it should be worth exploring what factors are (also) prognostic for complications in future work.

As a conclusion, routinely collected hospital data are potentially useful for PF research. Researchers and clinicians should consider exploiting such data for that purpose. In attempting to identify clinically relevant PFs for a variety of outcomes, PF research should differentiate between distinct treatments. Patients and clinicians could benefit from our findings in various ways, mainly through inclusion of the identified PFs in condition-specific prognostic models and using the results for (automated) internal feedback on outcomes and costs. In turn, this might support shared decision-making and may assist clinicians to determine which patients to monitor more closely after surgery.

## Methods

### Study design, setting and participants

A retrospective multicenter cohort study was performed using prospective routinely collected data retrieved from the ‘Benchmark Database’ serviced by LOGEX, a Dutch healthcare data analytics company. The data contain patient-level information on diagnosis, care activities and discharges, complemented by several patient characteristics. These data are primarily generated and used for reimbursement purposes and are considered an accurate source for research into the quality and costs of healthcare^5,22,23^. By using this database, we extracted data on four treatments for which surgical intervention was performed within a two-year period (2016–2017): laparoscopic resection of colorectal carcinoma (CRC), transurethral resection of urinary bladder carcinoma (UBC), acute percutaneous coronary intervention (aPCI), and total knee arthroplasty for osteoarthritis (TKA). We hypothesized that the inclusion of a diverse set of treatments in terms of disease burden, complexity, and acuteness would allow us to examine potential overlap between the cohort and underlying treatment-specific subgroups. We therefore aimed to best capture the abovementioned medical diversity while selecting treatments: CRC (complex, relatively high disease burden), UBC (medium complex, high disease burden), aPCI (acute intervention) and TKA (low complex, low disease burden). Follow-up was possible up to one year after the date of surgery. No ethical approval was required because patient data in the database was already fully anonymized.

### Outcomes and candidate prognostic factors

In selecting outcomes, we aimed to best capture all dimensions of treatment^24^. These dimensions can be divided into three tiers, each often representing different interests for patients. To summarize, tier 1 is achieved/retained health status, tier 2 indicates time to recovery and treatment disutility, and tier 3 indicates the sustainability of health or iatrogenic effects. Based on our data, this resulted in the inclusion of five outcomes in this study: in-hospital mortality (tier 1), intensive care unit (ICU) admission (tier 2), length of stay (post-surgery, tier 2), 30-day readmission (tier 3) and 30-day reintervention (tier 3).

In addition, we included in-hospital costs as an outcome, because of its clear relation to affordable and accessible healthcare^8^. All costs (i.e., surgical, diagnostic, clinic, and outpatient) incurred in the hospital with respect to the treatment undergone were included. Following the Dutch manual for costing studies, the total costs per treatment was defined as the sum over all delivered care activities multiplied by unit price per care activity^25^.

Based on previous PF research that identified patient factors as being (potentially) prognostic for our outcome variables^7,26^ and given data availability, we selected six candidate PFs. Patient age (in years), sex and socio-economic status (from highest (SES1) to lowest (SES3)) based on average income of the neighborhood in which patients lived at were readily available in the data. The number of hospitalizations in the year prior to treatment (all-cause, so not necessarily related to the conditions in the period of our current study), total days of spent in hospital in the year prior to treatment (again regardless of cause), and the Elixhauser Comorbidity Score (ECS) were computed using patient-specific care activities, diagnoses and disease history. The ECS is a graded point system that takes into account the severity of comorbidity, instead of solely including a collection of binary (comorbidity yes/no) scores^27^. The ECS was derived as a unique score for each included patient by attributing the corresponding Elixhauser Comorbidity Index Score to all known comorbidities that patients had at the time of treatment.

### Statistical analysis

Multivariable random-effect logistic and linear regression analysis were used to examine the association between our candidate PFs and the six outcomes (including costs). Specifically, separate regression models were developed for each combination of treatment and outcome (e.g., readmission for TKA patients), as well as separate models per outcome for the cohort. The estimated association for a candidate PF was adjusted for the effect of all other (candidate prognostic) factors because of potential confounding for the factor in question. For dichotomous outcomes, Firth logistic regression was used when the number of events was very low (e.g. in-hospital mortality among TKA patients)^28^. Because between-hospital variation in outcomes may influence study results when based on data from all hospitals pooled together^29^, we included hospital random effects in all models. The costs variable was log-transformed prior to estimation. Therefore, the estimated coefficients from the models for this variable can be interpreted as the percentage change in costs following a 1 unit increase in the relevant PF. Statistical significance was assessed using a significance level of 5%.

Prognostic models were constructed using tenfold cross-validation. The discriminative ability was evaluated using the concordance (*c*) statistic for dichotomous outcomes. Corresponding confidence intervals were calculated using bootstrap. C-statistic values were interpreted as fair (0.7–0.8) , good (0.8–0.9) or excellent (≥ 0.9)^30^. The models’ predictive accuracy was evaluated using the Brier score (range 0 = perfect and 0.25 = non-informative) for dichotomous outcomes^31^ and R-squared (proportion of explained variance) for continuous outcomes (i.e. LoS and costs). All analyses were conducted in R, version-3.6.3.

### Ethical statements

There were no experiments involved in this study and therefore approval of experimental protocols did not apply. An anonymous database was built from existing reimbursement data that was accumulated by hospitals under the Dutch Healthcare Law (Nederlandse Gezondheidswet). Since this study was based on legally obtained existing and anonymously processed data, no additional informed consent was required because there was no additional data collection. All methods were carried out in full accordance with privacy regulations and guidelines.

## Supplementary Information

Supplementary Information.

## Data Availability

This study brought together existing data obtained upon request and subject to license restrictions from several different sources. The database is not publicly available due to the (commercially, politically, ethically) sensitive nature of the data. No source consented to their data being retained or shared. Permission was acquired from a third party for use of the data in this study and following publication of this paper.
