# Supplementary material for: Identifying prognostic factors for clinical outcomes and costs in four high-volume surgical treatments using routinely collected hospital data
Source: Sci Rep. 2022 Apr 7;12:5902. doi: 10.1038/s41598-022-09972-6 (PMC8989991; doi:10.1038/s41598-022-09972-6)
Supplement: Supplementary file 1 — Supplementary Information. [file 41598_2022_9972_MOESM1_ESM.docx]

**Appendix 1**

*Supplementary material* – Cohort results (n=92,671)

In-hospital mortality

Age (OR 1.05), ECS score (OR 1.04) and days spent in hospital in the prior year (OR 1.06) statistically associated with an increased risk of in-hospital mortality. By contrast, female sex (OR 0.68) negatively associated with this outcome.

ICU admission

ECS (OR 1.10), prior hospitalizations (OR1.36), days spent in hospital in prior year (OR 1.08) and SES3 (OR 1.11) statistically associated with increased ICU admittance risk.

By contrast, female sex (OR 0.71) associated with decreased risk of ICU admission.

30-day readmission

ECS (OR 1.10), prior hospitalizations (OR 6.92) and SES2 (OR 1.13) associated with an increased 30-day readmission risk. By contrast, female sex (OR 0.71) and days spent in hospital in prior year (OR 0.80) associated with decreased readmission risk.

30-day reintervention

ECS (OR 1.09) and prior hospitalizations (OR 1.68) statistically significant association with an increased risk of reintervention. By contrast, female sex (OR 0.48) showed the opposite effect for this outcome.

Length of stay

Female sex (*b* 0.17), prior hospitalizations (*b* 0.28) and days spent in hospital in the prior year (*b* 0.06) were statistically associated with longer LoS.

In-hospital Costs

Prior hospitalizations associated strongest with estimated average costs of 32% per additional hospitalization, followed by female sex (18% cost increase compared to men) and days spent in hospital in the year prior to treatment (6% cost increase per day).

Prognostic model performance

C-statistics for the cohort were in-hospital mortality (0.78), ICU admittance (0.78), readmission (0.77) and reintervention (0.74). R-squared values were 0.05 for Los, and 0.20 for costs.
